# Self-Perceived Infertility is Not Always Associated with Having Fewer Children: Evidence from German Panel Data

**DOI:** 10.1007/s10680-023-09692-1

**Published:** 2024-02-12

**Authors:** Arthur L. Greil, Desmond D. Wallace, Jasmin Passet-Wittig, Julia McQuillan, Martin Bujard, Michele H. Lowry

**Affiliations:** 1 Division of Social Sciences, Alfred University, Alfred, NY USA; 2 Family and Fertility Research Area, Federal Institute for Population Research, Wiesbaden, Germany; 3 Department of Sociology, University of Nebraska-Lincoln, Lincoln, NE USA

**Keywords:** Fertility, Gender, Self-perceived infertility, Life course perspective, Growth curve models

## Abstract

Proximate determinants theory considers infertility rates a risk factor for lower fertility rates, but the assumption that people who perceive infertility will have fewer children has not been tested. This study investigates the association of self-perceived infertility with the number of children people have had after 11 years. Infertility implies reduced chances of conception (rather than sterility), but people do not always consistently perceive infertility over time. If people who think they are infertile at one time can later report no infertility, then does self-perceived infertility necessarily lead to having fewer children? We answer this question by analyzing 11 waves of the German family panel (pairfam) data using negative binomial growth curve models for eight core demographic subgroups created by combinations of gender (men/women), parity (0/1+children), and initial age groups (25–27 and 35–37). Those who repeatedly perceived themselves to be infertile (three times or more) had fewer children than those who perceived themselves to be infertile once or twice in only four of eight gender by initial parity by age groups. Only in four groups did people who perceived themselves to be infertile once or twice have fewer children than those who never perceived themselves to be infertile in both the unadjusted and adjusted models. Thus, self-perceived infertility does not necessarily result in fewer children. Rather, the association depends upon life course context and gender.

## Introduction

Fertility research has a long tradition of acknowledging the relevance of infertility for fertility behaviors and outcomes. In his influential Proximate Determinants Theory, Bongaarts (1978) included “sterility” as an important component in the equation that estimates change in fertility rates over time. Recent trends toward delaying childbearing have heightened interest in the role of infertility for understanding fertility rates. Johnson et al. (2019) have called for better integration of fertility and infertility research, in order to provide a more comprehensive understanding of women’s reproductive experiences. Proximate Determinants Theory was aimed at explaining fertility rates and therefore focuses analyses at the population levels. The individual level of analysis is increasingly of interest to demographers in their attempt to specify the mechanisms linking infertility to completed fertility (c.f. Beaujouan et al., 2019; Breyer et al., 2010; Shreffler et al., 2016). People tend to perceive that they are infertile if an anticipated pregnancy does not occur; it therefore appears reasonable to expect that those who perceive themselves to be infertile will ultimately have fewer children than those who do not perceive themselves to be infertile. Because more people are delaying childbearing, and many countries have below replacement fertility rates, the question of the association of self-perceived infertility and accumulated number of children is extremely relevant for demographers.

The current study examines the relationship between self-perceived infertility and number of biologically related children^1^ over time for individuals interviewed annually for up to 11 years. We argue that the relationship between self-perceived infertility and number of children might be more complex than it at first appears. Whether or not self-perceived infertility is associated with fewer children may depend on the frequency of that perception across multiple years, as well as on gender, age at first observation, and initial parity. The short answer to the research questions this study aims to answer is: it depends. Our results demonstrate that self-perceived infertility can, but does not necessarily, result in fewer children across the life course. Under specific conditions, self-perceived infertility can be associated with a higher number of children, particularly among those who were already mothers at the start of the panel. Thus, the relationship between self-perceived infertility and number of children depends on the combination of these perceptions, as well as on initial age, initial parity, and gender.

## Background and Literature Review

### Delayed Childbearing and Fertility in Developed Countries

Our data come from Germany, which, like most developed societies, is characterized by very low fertility. The average total fertility rate (TFR) for 38 Organization for Economic Co-Development (OECD) countries was 2.84 in 1970 (OECD, 2021). It fell below 2.00 for the first time in 1987 and is now well below replacement level (1.61 in 2019). Since 1993 in the EU, the TFR ranges between 1.4 and 1.6 (1.53 in 2018). Since 1973, the TFR in Germany ranged between 1.2 and 1.6 (1.54 in 2019). Despite the low birth rate, the cultural expectation to bear and rear children has remained strong in Europe and most parts of the world.

Becoming a parent is a central life course goal for many people (Johnson-Hanks et al., 2011), and parents across different cultures indicate that they place a high value on children (Nauck, 2014). Most Europeans have a personal ideal of one or more children, with two the most common desired number (Testa, 2012). Around 20% of women born in the early 1970s, however, have experienced permanent childlessness, and others have fewer children than they desire, indicating the existence of a “fertility gap” (Kreyenfeld & Konietzka, 2017). For adults who want children, infertility is often experienced as a major goal blockage (Loftus & Andriot, 2012).

A key source of the fertility gap in industrialized countries is the increasing age at which women have first births. In OECD countries, the mean age at first childbearing for women rose from about 25 years old in the 1970s to around 30 years old in 2019 (OECD, 2019). There are limits, however, to how long childbearing can be delayed without becoming forgone (Morgan & Rackin, 2010). The loss of oocytes from the ovaries is a process that begins in utero and accelerates after age 30 (Liu & Case, 2011). Demographic evidence suggests that fecundity among women begins to decrease around age 25 and decreases more rapidly after age 35 (Leridon, 2008). Delayed childbearing thus decreases the window of opportunity for giving birth (Habbema et al., 2015; Huinink & Kohli, 2014), so that many women are now attempting to have a first child at an age when their fecundity is already diminished. Thus, because infertility increases with age, especially for women, the postponement of childbearing amplifies the relevance of infertility for understanding stability and change in fertility rates.

Many studies have examined the postponement effect on period total fertility rates (c.f. Bongaarts & Feeney, 1998; Sobotka, 2004) as well as the societal and economic factors related to postponement (c.f. Mills et al., 2011; Ní Bhrolcháin & Beaujouan, 2012). Delayed childbearing is, then, linked to age-related infertility, which results in greater rates of childlessness and more women who complete their reproductive years having fewer children than they desired (Schmidt et al., 2012). Although German fertility patterns are comparable to those of other Western European countries, rates of permanent childlessness are higher in Germany (Kreyenfeld & Konietzka, 2017). One reason for the higher rate of childlessness is the challenge of reconciling work and family because of the lack of childcare facilities in West Germany during most of the data collection period for the German Family Panel (pairfam) (Bujard, 2011). We know of no longitudinal studies, however, that simultaneously model sociodemographic factors related to self-perceived infertility and the association of self-perceived infertility with number of children.

Several economically developed countries have designed policies and funded interventions to increase support for childcare in the expectation that one result will be increased fertility rates. Public funding for childcare is just one example of a policy aimed at increasing fertility in low-fertility states (Bauerschuster et al., 2016; Mills et al., 2011). Other pro-fertility policies have included paid parental leave and direct transfers of cash to families with young children, in addition to increased availability of childcare (Bergsvik et al., 2020). Some of these policies have been directly aimed at infertility, either by encouraging women to have children earlier by making them more aware of the relationship between infertility and aging (Goodchild, 2018; Nakagawa, 2018; Piagiani, 2016) or by providing subsidized access to assisted reproductive technology (ART) (Machado & Danto-de-Gadeano, 2015; Schmidt, 2007).

### State of Research on the Association between Infertility Measures and Number of Children over the Life Course

Physicians define infertility as 12 months of regular, unprotected intercourse without conception (Zegers-Hochschild et al., 2017). The definition implies reduced chances of conception but does not necessarily indicate sterility. In Germany, the current prevalence of infertility among women of reproductive age is between 6.6 and 7.5% (Passet-Wittig et al., 2016). As noted above, the risk of infertility increases with age. Consequently, the risk that people will remain childless or below their desired family size increases with aging. The effect of postponement of births on permanent involuntary childlessness, however, is still unclear. Estimations using a micro-simulation in six European countries showed a “considerable” effect (te Velde et al., 2012), but the authors cautioned that this effect might be eliminated if usage of ART were to become more widespread. Other scholars point out that it is difficult to determine how much of the relationship between fertility postponement and fertility rates is due to age-related infertility and how much is due to other factors, such as a desire for fewer children or the fact that those who delay childbearing become accustomed to their childless state (Schmidt et al., 2012). These authors also cautioned that late childbearing is on the rise and that, therefore, the completed fertility of women born in the 1970s and 1980s is not yet known. Furthermore, cross-country comparisons do not show a clear negative association between age at first birth and number of children (Schmidt et al., 2012.)

Estimations of the effect of infertility on fertility rates are made more complex because infertility is not a stable trait of people over time (Passet-Wittig et al., 2020). Data from pairfam show that German adults perceive an ability to have a child naturally (most of the time) but that a substantial minority (~ 5%) experience periods during which they perceive an inability to procreate naturally (Passet-Wittig et al., 2020). Thus, panel data is necessary to capture *patterns* of self-perceived infertility over the life course.

Most studies of the relationship between infertility and number of children have focused on the use of ART and the question of whether the use of ART is associated with increased fertility rates (Lazzari et al., 2023; Leridon, 2017; Tierney, 2022). The relevance of ART to the question of the association of infertility and fertility rates is based on the assumption that people who have used reproductive technology are infertile and would likely have not conceived without ART. However, only a portion of people who perceive themselves to be infertile use ART.

Some evidence suggests that ART use can at least partially make up for declines in number of children caused by delayed childbearing (Dik et al., 2009; Habbema et al., 2009; Hoorens et al., 2007; Lazzari et al., 2021; Leridon, 2004; Schmidt, 2007; Sobotka et al., 2008). Passet-Wittig and Bujard (2021) argue that the availability of ART may lead some women to delay childbearing even further, giving rise to a “Medically Assisted Reproductive Technology Paradox”. Other researchers find that the increases in the birthrate from ART are canceled by increases in delaying conception (Machado & Danto-de-Gadeano, 2015; Ohinata, 2011). Studies of the impact of ART on fertility rates are mostly based either on simulations (Dik et al., 2009; Leridon, 2004) or on cross-sectional comparisons between countries or states (Machado & Danto-de-Gadeano, 2015; Ohinata, 2011; Schmidt, 2007). Sobotka et al. (2008) compare women with ART-births to other women who have given birth, but they do not compare the number of children among women who experience fertility problems to the number of children among women who did not experience fertility problems.

We know of only two studies that use the individual as the unit of analysis to investigate fertility intentions and outcomes by self-perceived infertility status (Beaujouan et al., 2019; Shreffler et al., 2016). In a study that primarily focused on the relationship between age and fertility intentions, Beaujouan et al. (2019) were surprised to find that realization of fertility intentions by having a live birth did not vary significantly between women who reported that they or their partners had fertility problems and those who did not report fertility problems. In a study of the relationship between infertility and fertility intentions, Shreffler et al. (2016) also found that perception of a fertility problem was not significantly related to number of births.

Both of these studies were longitudinal, but they used only two waves of data. In addition, both studies focused only on women. We are not aware of any studies that examined number of children among men who have perceived themselves to be infertile. It is thus not clear how perceiving a fertility problem is associated with completed fertility. The goal of the current study is to examine the relationship between the number of times people perceived themselves to be infertile over the eleven years of the study and the number of children people have had after 11 years. Before we proceed, however, it is necessary to consider what “self-perceived infertility” means and how its meaning might change over the life course.

### Self-perceived Infertility, Meeting Medical Criteria, and Number of Children

Measures of self-perceived infertility do not perfectly reflect measures of medically defined infertility. Studies of women of reproductive age have found that many women who meet medical criteria for infertility do not perceive themselves to be infertile (Abbey et al., 1994; Loftus, 2009; Passet-Wittig et al., 2016; White et al., 2006). Conversely, women may self-identify as having a fertility problem even if they do not qualify as infertile by the medical definition (Greil et al., 2014). The imperfect association of self-identifying and meeting medical criteria has been found to be the case in a number of studies of younger women (Gemmill, 2018; Gemmill & Cowan, 2021; Gemmill et al., 2021; Polis & Zabin, 2012). Among the reasons young women gave for believing that they are infertile include a statement from a doctor, not becoming pregnant after having had sex without effective contraception, and knowing someone with fertility problems (Polis & Zabin, 2012).

Even though measures of self-perceived infertility do not perfectly map on to measures of medically defined infertility, the use of measures of self-perception as the sole measure of infertility is a common practice in survey research (Lazzari et al., 2022; Passet-Wittig et al., 2020). In a study using the NSFB, which includes measures of both self-perceived infertility and medically defined infertility, Lowry et al. (2020) found that 67% of the women responded consistently regardless of the measure used. Among those who did not give consistent responses, the vast majority (75%) met criteria but did not self-identify as infertile. Perceptions provide important information because people typically base their actions upon their definition of the situation (Thomas & Thomas, 1928). In the case of women without children (zero-parity), those aged 15–24 are most likely to have higher rates of self-perceived compared to medically defined infertility (Chandra et al., 2014). Lowry et al. (2020) showed that self-perceived infertility explains more of the variance in depressive symptoms than medically defined infertility. Thus, for studies primarily seeking to understand the lived experience and consequences of infertility, it is generally reasonable to rely upon survey questions that indicate whether people perceive themselves to be infertile (Lowry et al., 2020; McQuillan et al., 2022).

Our interpretation of the relationship of fertility perceptions and measures based on the medical definition is guided by core concepts and relevant insights from symbolic interactionism (McCall & Simmons, 1978; Mead, 1934) and identity control theory (Burke & Stets, 2009), a variant of symbolic interactionism. Self-perceptions can change over time as individuals interact with their social and physical environments (Burke & Stets, 2009; Goffman, 1959; Manzi et al., 2010). Identity control theory conceptualizes identity as a homeostatic process whereby individuals monitor implicit and explicit messages from their environments, compare this to their identity standard, and then adjust their behaviors to verify their identities (Burke & Stets, 2009).

In the case of infertility, symbolic interactionism suggests that self-perception might emerge if people might notice that their age peers are having babies, or if their parents ask when grandchildren might arrive. In addition, lack of conception is likely to be more salient for women than men because women gestate and give birth. If women think that their lives are not aligned with expectations that they perceive and accept, then they will experience a lack of homeostasis or alignment. Symbolic interactionism also suggests that people are likely to think of themselves as having a fertility problem, even without a medical diagnosis, if they want a child and are therefore more aware that they are not getting pregnant. Those who are explicitly trying to become pregnant are even more likely to perceive a fertility problem if conception does not occur in a timely manner. If they become pregnant relatively soon after perceiving a fertility problem, they are likely to cease to perceive a problem, but—if they continue to attempt to have a child without success—they will continue to perceive themselves to be infertile. People may sooner or later seek medical treatment, at which time doctors will define them as infertile, and this will likely help to solidify their self-perceptions (Greil et al., 2020).

It seems reasonable to suspect, then, that those who perceive themselves to be infertile in more years (e.g., three or more) will have fewer children than those never perceiving themselves to be infertile or perceiving themselves to be infertile in fewer years (e.g., one or two). However, identity control theory and symbolic interactionism suggest that people pay more attention to the lack of timely conception when they want to conceive, and that those trying to conceive may actually have more children in the long run. The possibility that those perceiving infertility may have more children is because people who perceive themselves to be infertile may actually conceive in a reasonable period of time (within a year), even though their desire for a child leads them to perceive themselves to be infertile. Those who are not trying to conceive or who do not want a child might not perceive themselves to be infertile but could still have a lower likelihood of conceiving.

Also, as we have seen, young women appear to be especially likely to report higher rates of self- perceived compared to medically defined infertility (Polis & Zabin, 2012). Unfounded self-perception of infertility can lead to problems such as less use of contraception and more unwanted pregnancies Data from the U.S. Veterans Affairs Healthcare System showed that—among women who do use contraception—those who perceive their susceptibility to pregnancy to be low are less likely to use the most effective methods compared to those with high perceived susceptibility (Britton et al., 2019). Over- (and under-) perception of infertility relative to actual ability to have a child, therefore, could complicate the presumed simple assumption that infertility leads to having fewer children.

## Method

### Sample

This study uses data from the German Family Panel *(*pairfam*)*, release 11.0, covering the years 2008/2009 to 2017/2018 (Brüderl et al., 2016; Huinink et al., 2011). Pairfam is a multidisciplinary study that consists of a nationwide representative sample of women and men living in Germany from three initial age groups based upon the years that they were born (age group 1 born in 1: 1991–1993, age group 2: 1981–1983, age group 3: 1971–1973). Data are collected yearly by computer-aided personal interviews. Modules which cover potentially sensitive topics such as infertility are conducted as computer-aided self-interviews. We use all waves up through wave 11. We do not use data from a complementary panel study (Demo-Diff) because it consists of East Germans only. As we are not interested in studying East Germans specifically, their overrepresentation could bias the coefficients of some variables.

The 11-wave data set (excluding Demo-Diff) contains 75,552 person-years (consisting of 17,419 people). Respondents from age group 1 (age 15–17 at first wave, person-years = 27,425, persons = 5543), and from age group 4, which was added only in wave 11 (age 5–7 at first wave, persons = 2476), were excluded because they would have no or very few infertility episodes.^2^ The remaining sample consists of 45,621 person-years (9,400 people). The analytical sample thus includes only women and men from age group 2 (age 25–27 at first wave) and age group 3 (35–37 years at first wave). We also excluded 3,027 people (13,953 person-years) who had two or more children at the time of the first wave, because most Germans have no more than two children, and the measure self-perceived infertility status requires information from all of the waves following the first. Thus, including those with two or more children would leave little room for an increase in the accumulated number of children over 11 waves of data.

These inclusion criteria reduced the sample to 31,668 person-years (6,373 people). In addition, we excluded 67 people (539 person-years) who had been sterilized at any wave, 132 people (422 person-years) who reported having a same-sex partner, and 1 person (6 person-years) who had a sex change operation, leaving a sample of 30,705 person-years (6213 people). The sample was reduced by an additional 63 person-years (16 people) due to missing data on any variables in the analysis. Thus, very few data points were lost due to listwise deletion of missing data. Also, we excluded the 679 people in age group 2 who were added as part of the Wave 11 refreshment sample. The refreshment sample provides only one wave of data; thus, it is impossible to observe change over time or to calculate a history of self-perceived infertility. Finally, we excluded 2,218 individuals for whom less than three waves of data were available because the measure of self-perceived infertility required at least three waves of data. The analytic sample consists of 27,124 person-years (3300 people) of which women contribute 13,588 person-years (1.647 people) and men contribute 13,536 person-years (1683 people). The panel data is unbalanced; therefore, gaps in individual panels due to unit-nonresponse may exist.

### Concepts and Measures

The dependent variable is a count of the number of children reported in a particular wave and ranges from 0 to 5.^3^ The focal independent variable, self-perceived infertility, is based on the question: “Some people are not able to conceive a child or to procreate naturally. As far as you know, is it physically possible for you to conceive a child or to procreate naturally?” Answer options were “definitely yes,” “probably yes,” “probably not,” “definitely not,” “don’t know,” and “I don’t want to answer that.” This question was asked in all eleven waves. For the multivariate analysis, we constructed a binary indicator in which those who chose “probably not” or “definitely not” were coded as perceiving themselves to be infertile and those who chose “definitely yes” or “probably yes” as perceiving fertility. Women who were pregnant and men whose partners were pregnant at the time of the interview were placed in the perceiving fertility category. We forgo making finer gradations because of the small number of cases in each of the categories that indicated a problem in a wave. For the same reason we exclude “don’t know” answers in the analyses.

We aggregated information across waves and constructed a three-category measure characterizing each person as (1) never perceived self to be infertile, (2) perceived self to be infertile once or twice, and (3) perceived self to be infertile three times or more. The resulting variable was treated as a time-invariant characteristic of individuals. We used “perceived self to be infertile once or twice” as the reference category to simplify the interpretation of results. Treating self-perceived infertility as a person-level variable leaves open the possibility that having a child could precede rather than follow the first episode of self-perceived infertility. We compared the timing of having the first child to the timing of the first episode of self-perceived infertility and determined that self-perceived infertility preceded having children in over 94% of cases. Although our approach is not perfect, it appears to be the best way to address our particular research question. We discuss this issue in more detail in the next section.

We included marital status, type of residence, place of birth, and level of education as control variables because these variables are relevant to cultural norms related to the number of children people have. Ever-married is a binary categorical indicator that has a value of “1” if the respondent was married at any time during the 11 waves of data collection and a value of “0” if the respondent was never married during the 11 waves of data collection. Type of residence is a categorical variable indicating whether the respondent lived in an urban area (reference category) during all waves, lived in both urban and rural areas at different waves or lived in a rural area for all waves of data collection,

Place of birth is a categorical variable that differentiates between persons born in West Germany, East Germany, and persons born outside of Germany (immigrants). We used West Germany, the largest group, as the reference category. This variable was measured at Wave 1 or at the first wave of data available if data from Wave 1 was missing. The measure of educational level was based on the International Standard Classification of Education (ISCED-97). “Low level of education” comprises those without a degree or lower secondary education (ISCED 1–3), “medium level of education” includes those with upper secondary (general and vocational) and postsecondary non tertiary education (ISCED 4–6), and “high level of education” includes those with tertiary education (ISCED 7–8). Education is measured at wave 11 or at the last wave of data available if data from Wave 11 was missing. If someone was still enrolled in school at their last wave of data collection, the measure assumes that the person will attain the corresponding degree. We treated this variable as categorical and used “low” as the reference category.

### Plan of Analysis

The goal of the analysis was to determine whether the accumulation of the number of children over eleven waves was associated with frequency of perception of a fertility problem (never perceived self to be infertile, perceived self to be infertile once or twice, perceived self to be infertile three or more times). The dependent variable, number of children, was a count variable; we therefore estimated the models using negative binomial regression with robust standard errors to estimate the growth curves. We considered other analytical approaches. Event history, survival, and hazard analyses are more suited to questions about the time to events, such as completed fertility (Allison, 2014). Fixed effects panel models are appropriate for questions about within person change in self-perceived infertility status and associations with changes in the outcome (Allison, 2009). Our interest, however, is in differential number of children by the end of the study by frequency of self-perceived infertility over the course of 11 years. We concluded that the growth curve approach is most appropriate for investigating whether infertility perception categories, a person characteristic, was associated with the pattern of the accumulation of children. We modeled time (1–11 years of annual data) as a polynomial to assess whether the change in the number of children is nonlinear (e.g., more rapid at first and then tapering at older ages). We conducted sensitivity analyses to see if using other methods of calculating standard errors would substantively affect our results and found that this was not the case.

One advantage of the pairfam data set is that both men and women are included in the sample, so that we are not limited to studying women only. Given that temporal fertility patterns likely vary by gender, we followed common practice and ran separate analyses for men and women. Because growth curves are likely to vary by initial parity, we ran separate analyses by parity. Growth curves are also likely to vary by age, so we ran separate analyses by initial age groups. All in all, we conducted separate analyses for the eight subgroups that result from the possible combinations of these variables (gender *x* parity *x* age group).

**Fig. 1:**
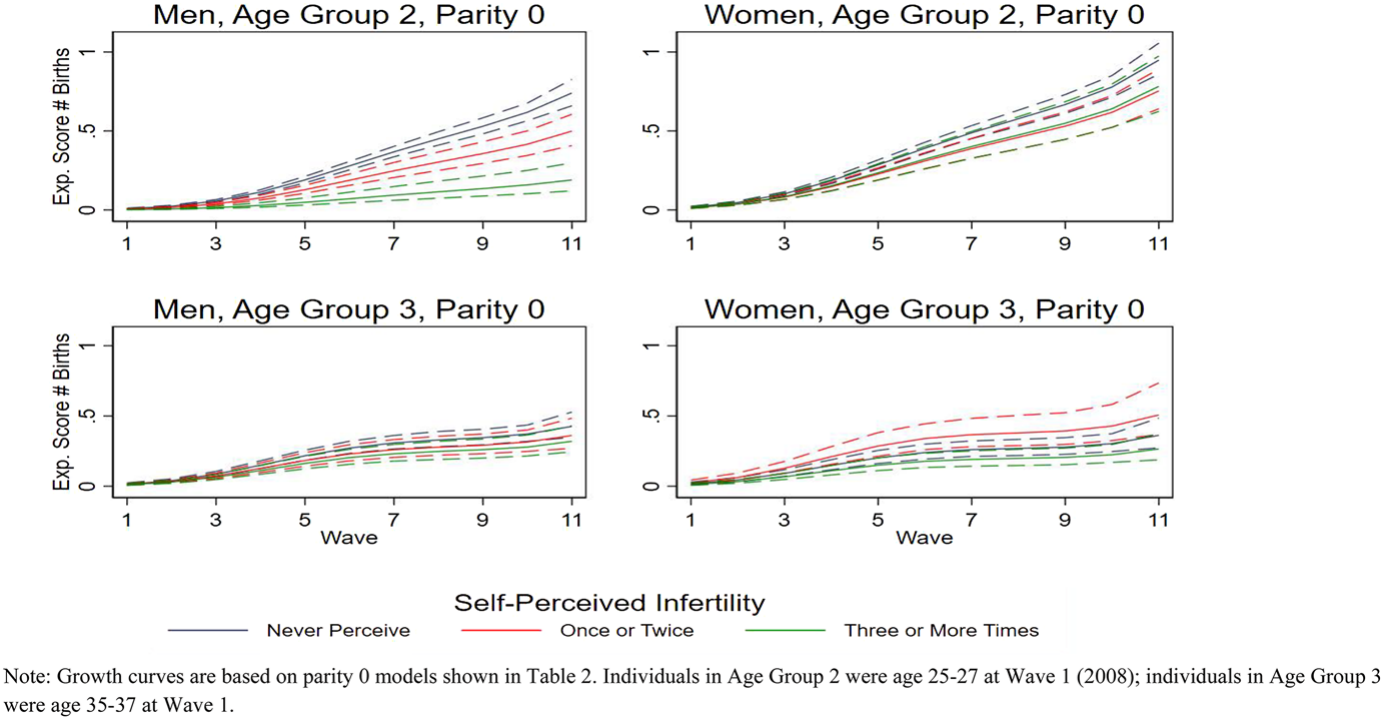
Growth curves for the expected number of children over 11 years by perceived infertility categories, parity 0 at first interview, and age group. *Note* growth curves are based on parity 0 models shown in Table 2. Individuals in age group 2 were age 25–27 at Wave 1 (2008); individuals in age group 3 were age 35–37 at Wave 1

**Fig. 2:**
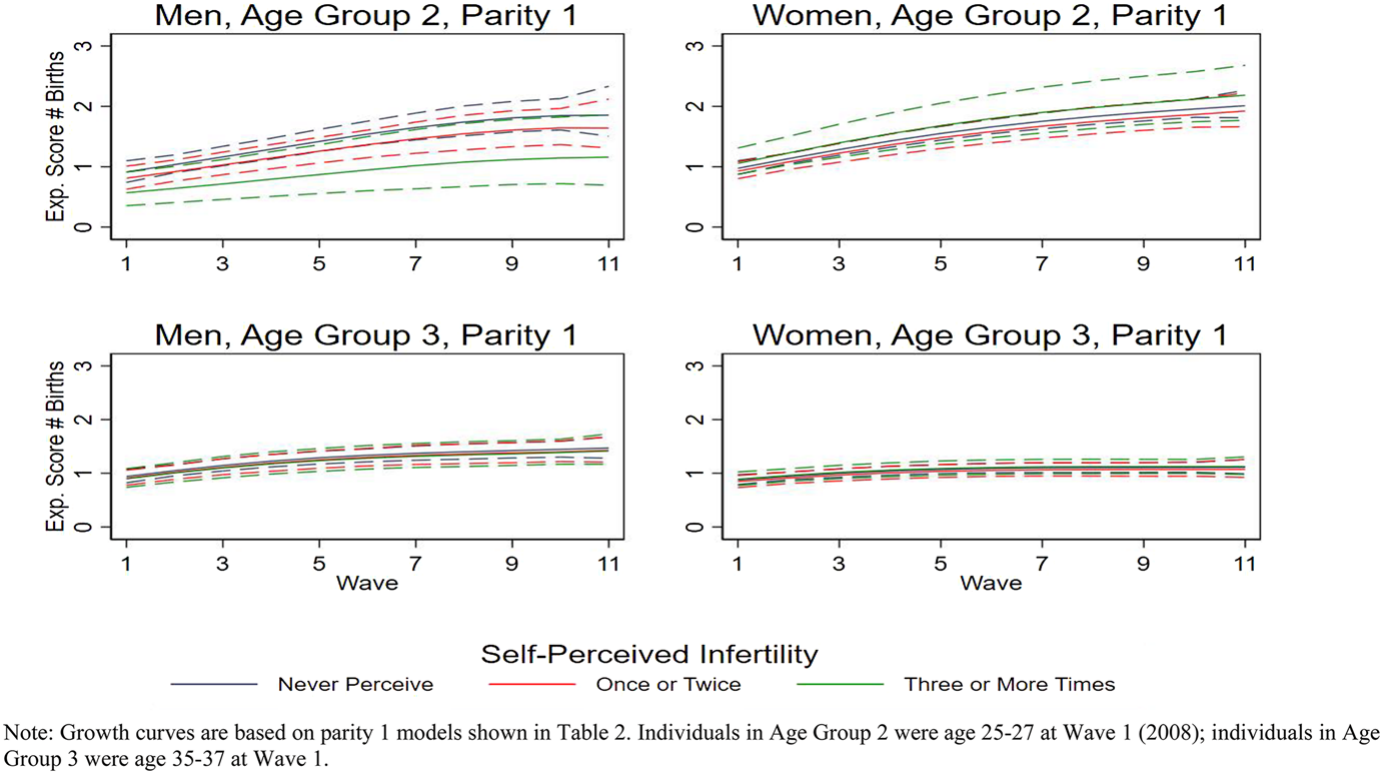
Growth curves for the expected number of children over 11 years by perceived infertility categories, parity 1 at first interview, by age group. *Note* growth curves are based on parity 1 models shown in Table 2. Individuals in age group 2 were age 25–27 at Wave 1 (2008); individuals in age group 3 were age 35–37 at Wave 1

It is not possible for the cumulative number of children to go down over time. Thus, our concern is with documenting differences in rate of increase in number of children among the three categories of frequency of self-perceived infertility over time. We estimated 24 growth curves (3 categories of self-perceived infertility times 8 separate group analyses); some of the growth curves were estimated based on a relatively small number of cases and therefore have fairly large standard errors. A table showing the number of cases that informed each growth curve can be found in Appendix. Figures 1 and 2 show the expected number of children (adjusted for control variables) by wave, age group, parity, and gender status. The confidence intervals for the graphs were estimated in R based on multiple simulations using the Zelig statistical program (Imai et al., 2008).

## Results

Table 1 provides descriptive statistics for the subsamples by gender and age group. Most men and women never perceived themselves to be infertile, and—as expected—men and women who started the study at younger ages (i.e., 25–27 years old) were less likely to report perceiving themselves to be infertile than those who were older when they started (i.e., 35–37 years old). A little over two-thirds of respondents had no children at the time of the first interview. About a quarter of respondents were married at some point during the eleven waves. Men and women in the older age group were about twice as likely to have ever been married as those in the younger age group. More older respondents had a child at the time of the first interview than younger respondents. Almost three quarters of the sample resided in urban areas during all the waves in which they participated. Most (two-thirds) of the sample were also native Germans living in West Germany; one-fifth was from East Germany, and under 15% were migrants. Over half of respondents had a “medium” educational level, and a little over a third reported a “high” level of education. Descriptive statistics for the dependent variable, number of children, are summarized in Appendix 1.

**Table 1:** Descriptive statistics for time-constant variables

	Men (*N* = 1683)				Women (*N* = 1647)				Total	
	Age group 2		Age group 3		Age group 2		Age group 3			
	Freq	%	Freq	%	Freq	%	Freq	%	Freq	%
Never perceived self as infertile	914	88.9	508	84.5	917	89.8	467	74.2	2806	85.6
Perceived once or twice	107	8.8	80	9.8	83	7.8	87	12.9	471	9.4
Perceived three times or more	22	2.2	52	5.8	29	2.5	80	12.8	183	4.9
Parity 0 at 1st interview	934	89.8	369	58.1	756	81.9	246	52.0	2305	70.2
Parity 1 at 1st interview	109	10.2	271	41.1	273	18.2	372	48.0	1025	29.8
Evermarried	116	8.7	267	59.6	238	18.5	301	40.8	922	24.4
*Residence*										
Always urban	691	73.1	409	69.3	678	73.5	442	74.4	2220	73.2
Mixed	132	12.3	55	10.2	135	11.6	57	9.1	379	11.1
Always rural	220	14.7	176	15	216	15	119	16.4	731	15.8
*Migrant background*										
West Germany	703	68.5	457	81.0	655	64.9	438	75.6	2253	69.2
East Germany	221	21.1	138	20.9	240	21.0	111	14.1	710	19.4
First Generation	119	10.4	45	8.2	134	14.2	69	10.3	367	14.4
Education					4.9					
Low	93	46.5	39	6.6	62	47.2	35	4.8	229	6.1
Medium	522	45.6	344	52.3	538	47.9	334	50.6	1738	48.2
High	428	41.0	257	41.1	429	41.7	249	44.6	1363	35.7
Total	1043	100.0	1039	100.0	1029	100.0	618	100.0	3300	100.0

Data: Eleven waves of pairfam. Frequencies are unweighted; percentages are weighted. Frequencies represent individuals. Percentages may not add up to 100% due to rounding

Individuals in age group 2 were age 25–27 at Wave 1 (2008); individuals in age group 3 were age 35–37 at Wave 1

Table 2 presents results of the negative binomial growth curve regression analyses for parity 0 respondents, and Table 3 presents results of the negative binomial growth curve regression analyses for parity one respondents. We present both unadjusted models (including the dependent variable, focal independent variable, and the time variables only) and adjusted models (including control variables as well) for all eight analyses. Figures 1 and 2 illustrate the estimated changes in the number of children reported over the eleven waves, separate by age group, gender, initial parity, and self-perceived infertility status, adjusted for the control variables. The figures show that, over time, the number of children increases in all subgroups, but some groups have more steep, linear, or nonlinear shapes. The patterns differ somewhat by parity. For those with no children initially, the curves are flatter early on and then get a little steeper. For those with children, the curves are a little steeper at the beginning and then level off later on. We next explore the growth curves for the subgroups in more detail.

**Table 2:** Negative binomial regression of number of children by gender, age group, parity 0 at first interview

	Men						Women					
	Unadjusted model			Adjusted model			Unadjusted model			Adjusted model		
	Coef	SE	P	Coef	SE	P	Coef	SE	P	Coef	SE	P
*Age group 2*												
Perceived infertility (ref = once or twice)												
Never perceived	**0.42**	**0.10**	**0.000**	**0.40**	**0.11**	**0.000**	0.05	0.08	0.554	**0.21**	**0.08**	**0.007**
Three or more times	− **0.88**	**0.29**	**0.002**	− **0.99**	**0.28**	**0.000**	0.00	0.14	0.989	0.00	0.13	0.994
Wave	**1.26**	**0.11**	**0.000**	**1.25**	**0.11**	**0.000**	**1.12**	**0.09**	**0.000**	**1.13**	**0.08**	**0.000**
Wave squared	**− 0.13**	**0.02**	**0.000**	**− 0.13**	**0.02**	**0.000**	**− 0.13**	**0.02**	**0.000**	**− 0.13**	**0.02**	**0.000**
Wave cubed	**0.01**	**0.00**	**0.000**	**0.01**	**0.00**	**0.000**	**0.01**	**0.00**	**0.000**	**0.01**	**0.00**	**0.000**
Ever married				**0.94**	**0.06**	**0.000**				**0.98**	**0.04**	**0.000**
Residence (ref = always urban)												
Mixed				**0.32**	**0.06**	**0.000**				− 0.07	0.06	0.257
Always rural				**0.50**	**0.05**	**0.000**				**0.28**	**0.05**	**0.000**
Migration background (ref = West Germany)												
East Germany				**0.14**	**0.06**	**0.010**				**0.26**	**0.05**	**0.000**
Migrant				**0.23**	**0.07**	**0.001**				**0.31**	**0.06**	**0.000**
Education (ref = low)												
Medium				− 0.02	0.11	0.849				0.04	0.12	0.754
High				**− 0.02**	**0.11**	0.856				– 0.02	0.12	0.886
Constant	− 5.123	0.205	0.000	− 5.40	0.22	0.000	− 3.87	0.16	0.000	− 4.38	0.19	0.000
*R* square	0.162			0.189			0.155			0.200		
*Age group 3*												
Perceived infertility (ref = once or twice)												
Never perceived	0.05	0.10	0.622	0.16	0.10	0.116	− 0.06	0.12	0.613	**− 0.35**	**0.13**	**0.007**
Three or more times	− 0.06	0.17	0.721	**− 0.13**	**0.16**	**0.405**	**− 0.37**	**0.18**	**0.034**	**− 0.63**	**0.18**	**0.001**
Wave	**1.23**	**0.14**	**0.000**	**1.20**	**0.14**	**0.000**	**1.17**	**0.17**	**0.000**	**1.16**	**0.17**	**0.000**
Wave squared	**− 0.16**	**0.03**	**0.000**	**− 0.16**	**0.03**	**0.000**	**− 0.17**	**0.04**	**0.000**	**− 0.16**	**0.04**	**0.000**
Wave cubed	**0.01**	**0.00**	**0.000**	**0.01**	**0.00**	**0.000**	**0.01**	**0.00**	**0.000**	**0.01**	**0.00**	**0.000**
Ever married				**0.89**	**0.08**	**0.000**				**0.82**	**0.09**	**0.000**
Residence (ref = urban)												
Mixed				0.03	0.14	0.851				**0.25**	**0.13**	**0.056**
Always rural				− **0.49**	**0.10**	**0.000**				− **0.60**	**0.15**	**0.000**
Migration background (ref = West Germany)												
East Germany				0.17	0.10	0.107				− **0.72**	**0.20**	**0.000**
Migrant				**0.43**	**0.19**	**0.026**				− **0.70**	**0.19**	**0.000**
Education (ref = low)												
Medium				0.17	0.20	0.385				− 0.05	0.39	0.905
High				**0.42**	**0.20**	**0.032**				0.14	0.39	0.716
Constant	− 3.91	0.24	0.000	− 4.50	0.30	0.000	− 3.73	0.29	0.000	− 3.69	0.43	0.000
*R* square	0.081			0.121			0.065			0.104		

Data: Eleven waves of pairfam (2305 individuals, 18,739 person-years). Negative binomial regression with polynomial time function

Individuals in age group 2 were age 25–27 at Wave 1 (2008); those in age group 3 were age 35–37 at Wave 1. Bold = significant

**Table 3:** Negative binomial regression of number of children by gender, age group, parity 1 at first interview

	Men						Women					
	Unadjusted model			Adjusted model			Unadjusted model			Adjusted model		
	Coef.	SE	*P*	Coef.	SE	*P*	Coef.	SE	*P*	Coef	SE	*P*
*Age group 2*												
Perceived infertility (ref = once or twice)												
Never perceived	**0.14**	**0.03**	**0.000**	**0.13**	**0.03**	**0.000**	**0.06**	**0.02**	**0.010**	0.04	0.03	0.126
Three or more times	− **0.36**	**0.05**	**0.000**	− **0.37**	**0.06**	**0.000**	**0.12**	**0.05**	**0.019**	**0.12**	**0.06**	**0.029**
Wave	**0.13**	**0.03**	**0.000**	**0.13**	**0.03**	**0.000**	**0.16**	**0.02**	**0.000**	**0.17**	**0.02**	**0.000**
Wave squared	0.00	0.01	0.573	0.00	0.01	0.627	− **0.01**	**0.00**	**0.003**	− **0.01**	**0.00**	**0.002**
Wave cubed	0.00	0.00	0.775	0.00	0.00	0.692	0.00	0.00	0.131	0.00	0.00	0.118
Ever married				**0.09**	**0.03**	**0.000**				**0.15**	**0.02**	**0.000**
Residence (ref = always urban)												
Mixed				− 0.01	0.04	0.740				− 0.05	0.03	0.069
Always rural				− 0.03	0.03	0.380				− 0.01	0.02	0.590
Migration background (ref = West Germany)												
East Germany				0.03	0.03	0.333				− **0.12**	**0.02**	**0.000**
Migrant				**0.10**	**0.03**	**0.002**				− **0.08**	**0.02**	**0.000**
Education (ref = low)												
Medium				− **0.13**	**0.03**	**0.000**				**0.11**	**0.03**	**0.000**
High				− 0.05	0.04	0.242				**0.09**	**0.03**	**0.002**
Constant	− 0.11	0.03	0.000	− 0.07	0.04	0.082	− 0.07	0.02	0.004	− 0.16	0.04	0.000
*R* square	0.034			0.038			0.029			0.036		
*Age group 3*												
Perceived infertility (ref = once or twice)												
Never perceived	0.03	0.02	0.239	0.03	0.02	0.154	0.04	0.02	**0.020**	**0.03**	**0.02**	**0.036**
Three or more times	− 0.05	0.04	0.202	− 0.01	0.04	0.840	0.04	0.02	0.150	0.05	0.02	0.051
Wave	**0.12**	**0.02**	**0.000**	**0.12**	**0.02**	**0.000**	**0.09**	**0.01**	**0.000**	**0.09**	**0.01**	**0.000**
Wave squared	− **0.01**	**0.00**	**0.005**	− **0.01**	**0.00**	**0.003**	− **0.01**	**0.00**	**0.002**	**− 0.01**	**0.00**	**0.001**
Wave cubed	0.00	0.00	0.108	0.00	0.00	0.087	0.00	0.00	0.074	**0.00**	**0.00**	**0.047**
Ever married				**0.07**	**0.02**	**0.000**				**0.13**	**0.01**	**0.000**
Residence (ref = urban)												
Mixed				**0.12**	**0.03**	**0.000**				0.00	0.02	0.825
Always rural				**0.06**	**0.02**	**0.001**				**− 0.04**	**0.01**	**0.007**
Migration background (ref = West Germany)												
East Germany				− **0.09**	**0.02**	**0.000**				− .01	0.01	0.457
Migrant				**0.09**	**0.03**	**0.005**				− **0.07**	**0.02**	**0.000**
Education (ref = low)												
Medium				**0.11**	**0.03**	**0.001**				**0.06**	**.01**	**0.000**
High				**0.22**	**0.03**	**0.000**				**0.24**	**0.01**	**0.000**
Constant	− 0.02	.02	.449	− 0.23	0.04	.000	-.04	.02	.022	− 0.22	0.02	0.000
*R* square	0.012			0.017			0.003			0.010		

Data: Eleven waves of pairfam (1025 individuals, 8385 person-years). Negative binomial regression with polynomial time function

Individuals in age group 2 were age 25–27 at Wave 1 (2008); those in age group 3 were age 35–37 at Wave 1. Bold = significant

Of the eight subgroups, self-perceived infertility is associated with number of children at the conventional 0.05 level in six of eight groups in the adjusted models:

1. Among men from the younger age group, whether they were initially childless (i.e., parity 0) or not, eventual number of children is highest for those who never perceived themselves to be infertile and lowest among those who perceived themselves to be infertile three or more times. This relationship holds for both the unadjusted and adjusted analyses.
2. Among men from the older age group, whether they were initially childless (i.e., parity 0) or not, self-perceived infertility status was not associated with eventual number of children. This relationship holds for both the unadjusted and adjusted analyses.
3. Among women from the younger age group who were initially childless (i.e., parity 0), the unadjusted model shows no relationship between eventual number of children and self-perceived infertility status. In the adjusted model, however, it appears that those who never perceived themselves to be infertile eventually had more children than those who perceived themselves to be infertile once or twice as well as those who perceived themselves to be infertile three times or more.
4. In the unadjusted model for women from the older age group who were initially childless (i.e., parity 0), those who perceived themselves to be infertile three or more times had fewer children than those who perceived once or twice, but there was no difference among those who perceived once or twice and those who never perceived themselves to be infertile. In the adjusted model, eventual number of children was higher among those who perceived themselves to be infertile once or twice compared to both those who perceived themselves to be infertile three or more times and those who never perceived themselves to be infertile.
5. Counter to expectations, the unadjusted model shows that, among women from the younger age group who were initially mothers (i.e., parity 1), those who perceived themselves to be infertile three or more times and those who never perceived themselves to be infertile had a higher eventual number of children than those who perceived themselves to be infertile once or twice. The adjusted model still shows that women who perceived themselves to be infertile three or more times had a higher eventual number of children than those who perceived themselves to be infertile once or twice but that there was no longer a difference between those who never perceived themselves to be infertile and those who perceived themselves to be infertile once or twice.
6. Both the unadjusted and adjusted models show that, among women from the younger age group who were initially mothers (i.e., parity 1), those who never perceived themselves to be infertile had a higher eventual number of children than those who perceived themselves to be infertile once or twice. There was no difference between women who perceived themselves to be infertile three or more times and those who perceived themselves to be infertile once or twice.

Notably, there is no single pattern of association of self-perceived infertility and number of children that persists across subgroups. Those who repeatedly perceived themselves to be infertile (three times or more) had fewer children than those who perceived themselves to be infertile once or twice in only three of eight “gender by initial parity by age group” groups. Parity 1 women in the younger age group who perceived themselves to be infertile three times or more actually had more children than women who perceived themselves to be infertile once or twice. Only in four groups did people who perceived themselves to be infertile once or twice have fewer children than those who never perceived themselves to be infertile in both the unadjusted and adjusted models. Parity 1 women in the older age group who perceived themselves to be infertile once or twice had more children than comparable women who never perceived themselves to be infertile. Thus, the consequences of self-perceived infertility depend upon gender and reproductive life course situation.

We now turn briefly to other variables in the model. The linear measure for time (wave) is positive in all models for those initially childless, and for all but men in the younger age group (age group 2), who had one child at the start of the study. The association is nonlinear for all groups who had no children initially (Wave squared and wave cubed are statistically significant, and as the figures show, indicate “plateaus” over time.). Among those with initial parity 1, at least one of the nonlinear terms (wave squared and wave cubed) are significant, except for fathers in the younger age group. It was therefore necessary to use a polynomial time function to capture the nonlinear process of the accumulation of children.

Turning now to the control variables in the adjusted models, people in all subsamples who were ever married eventually had more children than those who were never married. In most analyses, people who lived in rural areas eventually had more children than people who lived in urban areas. Compared to those born in West Germany, persons born abroad have a higher number of children in most subsamples. The difference in eventual number of children between those from East and West Germany differed by gender, parity, and age group. The relationship between education and eventual number of children also differed by gender, parity, and age group.

## Discussion

In this article, we examine the relationship between self-perceived infertility and the pattern of number of children over time using a German population sample. This study contributes to answering the still open question of the potential impact of self-perceived infertility on the number of children people have. We estimated growth curves for the number of children by frequency of self-perceived infertility across 11 waves (never perceived self to be infertile, perceived self to be infertile once or twice, perceived self to be infertile three times or more). Because age, gender, and initial parity are core demographic characteristics associated with achieved number of children, we conducted separate analyses by age group (25–27 at wave 1 vs. 35–37 at wave 1), gender, and initial parity (0/1).

People who perceive themselves to be infertile often have children later in life; this article presents specific temporal patterns for having children or different levels of self-perceived infertility. “Common sense” suggests that women and men who repeatedly perceived themselves to be infertile (three times or more) would have fewer children than those who perceived themselves to be infertile once or twice, but we found this to be true in only three of eight analyses. Strikingly, parity 1 women in both age groups who perceived themselves to be infertile three times or more actually had *more* children than women who perceived themselves to be infertile once or twice. This finding could reflect a stronger commitment to having a child among these women (Shreffler et al., 2016). In two subgroups—men in the older age group—self-perceived infertility status was not associated with number of children. The latter finding could reflect small subsample sizes more than an actual null association, but the coefficients in these two groups are quite small compared to the coefficients in the subgroups with statistically significant associations.

Persons who perceived themselves to be infertile once or twice have fewer children than those who never perceived themselves to be infertile in four of eight analyses (adjusted models). In three of the other four analyses, women who perceived themselves to be infertile once or twice did not differ significantly in terms of number of children from those who never perceived themselves to be infertile. Women who were parity 0 and were in the older age group who perceived themselves to be infertile once or twice had more children than comparable women who never perceived themselves to be infertile. This suggests that it is not always the case that perceiving oneself to be infertile only once or twice is not necessarily linked to having fewer children. It is also possible that many of the women without children (parity 0) who started the study at ages 35–37 and never perceived infertility were actually voluntarily child-free and thus this group has an unusually low number of children. Among some groups, perceiving oneself to be infertile once or twice could indicate increased focus on having children and thus a higher awareness of problems procreating.

Finding considerable variation in the association of perceived infertility and number of children, including higher numbers of children among those perceiving infertility, has fundamental consequences for longitudinal survey data collection and for understanding demographic trends (e.g., impact of infertility on birth rates, increasing rates of late fertility). If longitudinal surveys assume that those who perceive themselves to be infertile will no longer have children, they might include skip patterns that fail to ask about birth intentions or desires. The results of the current study suggest that it is more accurate to characterize self-perceived infertility as a temporary state rather than a permanent trait (see also Passet-Wittig et al., 2020).

These results demonstrate that the relationship between self-perceived infertility and number of children depends on the accumulation of these perceptions over time and on different combinations of core demographic characteristics (e.g., initial parity, gender and age group). Identity control theory suggests mechanisms that are likely to contribute to having children among those who perceive themselves to be infertile, including making extra efforts to conceive (e.g., ceasing contraception, monitoring cycles, timing intercourse, or seeking medical help) or perceptions of infertility reflecting heightened attention to lack of conception following the cessation of contraception. Recognizing the heterogeneity among those who perceive themselves to be infertile could help explain why Beaujouan et al. (2019) did not find any differences in the realization of short-term fertility intentions between those who reported fertility problems and those who did not.

There are limitations in this study. As noted above, we calculated 24 growth curves (3 categories of self-perceived infertility times 8 separate analyses). Although pairfam starts with a large sample, some estimations, especially involving those who perceived themselves to be infertile three or more times, are based on a relatively small number of cases (see Appendix). Thus, results involving those who perceive three or more times should be regarded with caution, and conclusions regarding this group must be regarded as tentative. Some researchers might prefer to measure infertility using medical criteria rather than subjective perception because measures of perceived infertility do not perfectly reflect measures of medically defined infertility, but the relevance of subjective measures for behaviors associated with having children has been well documented.

Only some of the respondents in this study have completed their reproductive years, and some may go on to have (additional) children. Therefore, our focus was on the number of children people have rather than completed fertility. This is particularly relevant for respondents in the younger age group (who were 35–37 in the last wave), who have had fewer opportunities both to have children and to perceive themselves to be infertile. This is especially true for highly educated women who often postpone childbirth to ages above 37. It also should be noted that some parity 1 individuals may have perceived themselves to be infertile before having their first child. For women in the older age group, the analysis period covers the final reproductive years as these women are 45–47 in the last wave. Future studies on completed fertility with panel data longer than 11 waves are necessary to cover the full reproductive life span.

We did not include data on treatment for infertility. Information on fertility treatment is available in pairfam, but, for waves 1–7, only for those who actually perceive themselves to be infertile. Our conceptual model and empirical results suggest that receipt of treatment may well moderate the relationship between perception of a problem and number of children. We did not include this variable out of concern that it would complicate an already complex analysis. In this initial exploration, we wanted to determine whether self-perceived infertility consistently led to a lower number of children, which as the results suggest, is not the case. Furthermore, available pairfam data cannot tell us whether people have ever been diagnosed with a medical fertility problem. It would be interesting to see whether having a medical diagnosis moderates the relationship between self-perceived infertility and number of children.

It should also be noted that the question about self-perceived fertility problems provided little guidance as to what criteria respondents should use to determine whether they had a fertility problem. It is thus possible that different respondents may have interpreted this question differently. For example, some respondents may have interpreted the question as asking about themselves as individuals, while others may have interpreted it as asking about them as members of a particular couple. This may have been especially a problem for men as men do not become pregnant. It is unclear how men might answer this question if they have fathered children in the past, but their current partner has not become pregnant. Nonetheless—as noted above—there are good reasons to study self-perceived infertility. Self-perception measures are often the only measures available to us in surveys, and self-perceived infertility has been associated with both infertility-related behavior and well-being (Lowry et al, 2020; McQuillan et al., 2022). Thus, for studies primarily seeking to understand the lived experience and consequences of infertility, it is reasonable to focus on questions about perceptions even if we are not certain that all respondents will interpret them in the same way.

The complex patterns of relationships between self-perceived infertility and number of children which we observed challenge hitherto assumptions about the implications of (self-perceived) infertility. The assessment of own (in)fertility is a relevant element of fertility behavior in life course contexts with different possible directions of influence: In some cases, it can be associated with a lower future number of children and, in others, even a higher number. These differing temporal patterns and their underlying mechanisms are worthy of future study. The relevance of our work for demographers is clear: rather than assume that people who report self-perceived infertility will not have any more children (i.e., treating infertility in surveys as the “sterility” proximate determinant), fertility research should include these measures in their analyses as important information concerning fertility behavior over the life course.

## Appendix

Unadjusted number of children in final wave by perceiving oneself as infertile, gender, age group, and parity.

**Table:**

	Age group 2						Age group 3						All groups combined		
	Men			Women			Men			Women					
	Mean	SD	*N*	Mean	SD	*N*	Mean	SD	*N*	Mean	SD	*N*	Mean	SD	*N*
*Parity* 0															
Perceived self as infertile															
Never perceived	0.69	0.82	827	0.89	0.93	680	0.53	0.81	283	0.37	0.63	168	0.71	0.85	1958
Perceived once or twice	0.42	0.70	87	0.84	1.05	56	0.53	0.84	50	0.42	0.65	34	0.55	0.83	227
Three or more times	0.16	0.49	20	0.84	1.06	20	0.46	0.91`	36	0.30	0.69	44	0.41	0.82	120
All self-perception statuses	0.65	0.81	934	0.89	0.94	756	0.52	0.82	369	0.36	0.65	246	0.67	0.85	2305
*Parity* 1															
Perceived self as infertile															
Never perceived	1.88	0.76	87	1.89	0.78	237	1.50	0.62	225	1.21	0.49	283	1.58	0.68	832
Perceived once or twice	1.58	0.60	20	1.73	0.72	27	1.57	0.63	30	1.19	0.38	53	1.45	0.59	130
Three or more times	1.00	0.00	11	2.17	0.78	9	1.47	0.63	16	1.24	0.48	36	1.40	0.64	63
All self-perception statuses	1.80	0.74	118	1.88	0.77	273	1.50	0.62	271	1.24	0.42	372	1.54	0.67	1025

*N* = 3330; mean = 0.93; SD = 0.90. Individuals in age group 2 were age 25–27 at wave 1 (2008); those in age group 3 were age 35–37.

## Footnotes

1 Hereafter referred to simply as “children.” We use the term “number of children” because we use data from a panel study of women and men who may still have additional children after the observation period.

2 The question on perceived infertility was asked only to respondents age 21 and older.

3 We used the pairfam constructed variable “nbiokids,” which includes children biologically related to the respondent, as the dependent variable.
